# Multidisciplinary management of difficult/aggressive growth-hormone pituitary neuro-endocrine tumors

**DOI:** 10.3389/fendo.2023.1123267

**Published:** 2023-05-03

**Authors:** Antonio Bianchi, Sabrina Chiloiro, Antonella Giampietro, Simona Gaudino, Rosalinda Calandrelli, Ciro Mazzarella, Carmelo Caldarella, Mario Rigante, Marco Gessi, Liverana Lauretti, Laura De Marinis, Alessandro Olivi, Alfredo Pontecorvi, Francesco Doglietto

**Affiliations:** ^1^ Pituitary Unit, Department of Medical and Surgical Sciences, Fondazione Policlinico Universitario Agostino Gemelli, Istituto di Ricovero e Cura a Carattere Scientifico (IRCCS), Rome, Italy; ^2^ Endocrinology and Diabetes Unit, Department of Medical and Surgical Translational Sciences, Università Cattolica del Sacro Cuore, Fondazione Policlinico Universitario Agostino Gemelli, Istituto di Ricovero e Cura a Carattere Scientifico (IRCCS), Rome, Italy; ^3^ Radiology and Neuroradiology Unit, Department of Imaging, Radiation Therapy and Hematology, Università Cattolica del Sacro Cuore, Fondazione Policlinico Universitario Agostino Gemelli, Istituto di Ricovero e Cura a Carattere Scientifico (IRCCS), Rome, Italy; ^4^ Radiation Therapy Unit, Department of Imaging, Radiation Therapy and Hematology, Fondazione Policlinico Universitario Agostino Gemelli, Istituto di Ricovero e Cura a Carattere Scientifico (IRCCS), Rome, Italy; ^5^ Nuclear Medicine Unit, Department of Imaging, Radiation Therapy and Hematology, Fondazione Policlinico Universitario Agostino Gemelli, Istituto di Ricovero e Cura a Carattere Scientifico (IRCCS), Rome, Italy; ^6^ Department of Aging, Neurological, Orthopedic and Head-Neck Sciences, Fondazione Policlinico Universitario Agostino Gemelli, Istituto di Ricovero e Cura a Carattere Scientifico (IRCCS), Rome, Italy; ^7^ Neuropathology Unit, Fondazione Policlinico Universitario Agostino Gemelli, Istituto di Ricovero e Cura a Carattere Scientifico (IRCCS), Rome, Italy; ^8^ Pathology Unit of Head and Neck, Lung and Endocrine Systems, Università Cattolica del Sacro Cuore, Fondazione Policlinico Universitario Agostino Gemelli, Istituto di Ricovero e Cura a Carattere Scientifico (IRCCS), Rome, Italy; ^9^ Neurosurgery Unit, Department of Neurosciences, Fondazione Policlinico Universitario Agostino Gemelli, Istituto di Ricovero e Cura a Carattere Scientifico (IRCCS), Rome, Italy; ^10^ Neurosurgery Unit, Department of Neurosciences, Catholic University School of Medicine, Rome, Italy

**Keywords:** aggressive pituitary adenoma, acromegaly, growth hormone, multidisciplinary, pituitary adenoma, pituitary neuro-endocrine tumor

## Abstract

Growth Hormone-secreting adenomas exhibits variable biological behavior and heterogeneous natural history, ranging from small adenomas and mild disease, to invasive and aggressive neoplasms with more severe clinical picture. Patients not cured or controlled after neurosurgical and first-generation somatostatin receptor ligands (SRL) therapy could require multiple surgical, medical and/or radiation treatments to achieve disease control. To date, no clinical, laboratory, histopathological, or neuroradiological markers are able to define the aggressiveness or predict the disease prognosis in patients with acromegaly. Therefore, the management of these patients requires careful evaluation of laboratory assessments, diagnostic criteria, neuroradiology examinations, and neurosurgical approaches to choose an effective and patient-tailored medical therapy. A multidisciplinary approach is particularly useful in difficult/aggressive acromegaly to schedule multimodal treatment, which includes radiation therapy, chemotherapy with temozolomide and other, recent emerging treatments. Herein, we describe the role of the different members of the multidisciplinary team according to our personal experience; a flow-chart for the therapeutic approach of difficult/aggressive acromegaly patients is proposed.

## 1 Introduction

Growth hormone (GH) secreting pituitary adenomas or pituitary neuroendocrine tumors (PitNET) represent a heterogeneous group of neoplasia with complex and variable biological behavior. Although surgery and first-line medical therapy with somatostatin receptor ligands (SRL) are the cornerstones of treatment of acromegaly, a non-negligible percentage of patients (from 24 to 65%) do not reach biochemical disease control (1).

The inadequate biochemical control of acromegaly may be due to suboptimal dosing of medical therapies, poor compliance to treatments, resistance to drugs, tumor phenotype, inadequate monitoring, and uncertainty of GH and IGF-I assays (2). For these reasons, in this paper we defined “difficult” the GHomas not cured/controlled after neurosurgical (first line) and first generation SRL (second line) therapy. Concerning the term “aggressive”, at present, a clear definition of aggressive pituitary adenomas remains equivocal (3). According to clinical practice (4) and to European Society of Endocrinology (ESE) guidelines (5), GH-PitNET/adenomas are defined as aggressive if, invasive, with a high proliferative index, with refractory behavior and poor response to optimal standard treatments such as surgical, medical, and radiotherapy, and in cases of multiple local recurrences (5, 6). In our opinion, the most reliable definition is that resulting from cluster analysis (type 3 acromegalic patients) defined by Cuevas-Ramos and coworkers (4) and based on clinical, radiological, histopathological, and outcome characteristics. This definition of difficult/aggressive ranges between SRL partial responders adenomas to more aggressive and invasive one and it is a good compromise between two evidences: the definition of aggressiveness according to the presence of local invasion of surrounding structures of about 35-50% of these neoplasms based on the recent validation of the French five-tiered classification (7–11); the definition of aggressiveness based on the ESE guidelines: “the hallmark of aggressiveness is clinically relevant tumour growth despite the use of optimal standard therapies, which entails a combination of medical therapies, surgery and radiotherapy” (5).

The incidence of aggressive adenomas/PitNET ranges from 4.5 to 31% of patients and reflect the different definitions (12). A comprehensive definition of difficult/aggressive GH-secreting PitNETs probably requires multidisciplinary evaluation in a team that includes experts in all the fields of pituitary disease, including neuro-endocrinology, neurosurgery, neuropathology, neuroradiology, otolaryngology, radiation oncology and nuclear medicine (13).

In this perspective viewpoint, we critically review the different aspects that should be taken into account for multidisciplinary management of a patient with a difficult/aggressive GH-secreting adenomas/PitNET from diagnosis to the choice of treatment.

## 2 Clinical and biochemical diagnosis of aggressive GH-PitNETs

Early recognition of severe disease is crucial. At the time of diagnosis, clinical criteria for identifying a difficult/aggressive case of GH-secreting PitNETs are not univocally recognized. In clinical practice, before the treatment choice, we suspect difficult/aggressive GH-secreting adenomas/PitNET typically in cases of young patients, who might also refer signs and symptoms of hypopituitarism rather than those related to GH/IGF-I excess. This high prevalence of pituitary dysfunction in young acromegaly patients may be explained by the presence of an invasive macro- or giant adenomas/PitNET with a extrasellar extension and/or with an unusually high rate of growth (5, 14). In parallel, high GH and IGF-I levels at diagnosis are an expression of larger and invasive adenomas/PitNET (4). No data are available on comorbidities that can be identified after the diagnosis of acromegaly. However, the presence of multiple acromegaly-related complications in the same patient is considered as suggestive of more severe disease (15), due to a direct effect of high levels of circulating GH/IGF-1 (16) or to the diagnostic delay.

## 3 Radiological evaluation

Magnetic resonance imaging (MRI) and computed tomography (CT) can provide significant data that should alert the clinician to a potentially aggressive behavior of the GH-secreting PitNET. Large tumor size, extra-sellar extension, and postoperative residues are generally considered to be predictors of poor outcome. A diameter that is > 15 mm has been reported in aggressive GH tumors (12) and has been detected mostly in sparsely granulated lesions (17). Aggressive GH-adenomas/PitNET show specific patterns of growth. Unlike other PitNETs, infrasellar invasion is the most common pattern of growth, with erosion of the sellar floor and clivus, that are commonly detected on preoperative CT (18, 19). The invasion of dura and sellar diaphragm is poorly identified by MRI (20). The major site of dura invasion is the medial wall of the cavernous sinus.

The tumor’s relation to the cavernous sinus is classically quantified by Knosp scoring on MRI, which measures lateral tumor extension in relation to the internal carotid arteries preoperatively (21, 22). If compared to densely granulated (DS) GH-secreting PitNETs, sparsely granulated (SG) tumors are more likely to invade the cavernous sinus (grades 3–4 of Knosp’s classification) (17).Some MRI sequences and techniques may help to predict the more SG phenotype of GH secreting PitNET: high T2 signal intensity, due to a low collagen content and low number of secreted granules, and a more avid enhancement are found in SG adenomas (17). T2WI-based texture parameters of the whole tumor appear to be able to provide more quantitative information and help predict granulation pattern better than T2 signal intensity (23). Preliminary studies showed that texture signatures based on T1WI and post-contrast T1WI of specific solid tumor areas may reflect on the biological behavior of the tumor and achieved greater diagnostic efficacy than in the entire tumor (24).

In the post-operative period and during follow-up, MRI plays a significant role in assessing surgical outcomes, as well as documenting local recurrence, progression of residual disease to surrounding tissues, and rare distant metastases both in the central nervous system and in extracranial organs.

Some pituitary carcinomas may develop from an invasive GH-adenomas/PitNET and metastasize *via* the subarachnoid space and lymph and blood vessels to the brain and extracranial organs, especially liver or bones, also requiring total body CT (25, 26).

## 4 Surgery

Surgical treatment of acromegaly presents specific challenges, including specific anesthesiologic issues, anatomical variations of the sellar and parasellar regions, and nasal mucosa edema (27), which need to be evaluated pre-operatively to optimize treatment and decrease complications. Endoscopic transsphenoidal surgery is a relatively novel technique, in which the endoscope, together with the optimization of the transnasal corridor, provides the possibility of visualizing even the extrasellar components of pituitary adenomas. This has led to the possibility of exploring the suprasellar area and cavernous sinus with limited morbidity and higher efficacy compared to “classic” surgical approaches (28–32). Surgical experience has been demonstrated to be a significant factor for optimal outcomes, underlining the importance of centers of excellence and sub-specializations (33, 34) Other technical advancements, such as intraoperative Doppler and neuronavigation, have also led to safer and more effective surgeries. Despite these recent technical and organizational advancements, true invasiveness in GH-adenomas/PitNET remains a major limiting factor for surgical disease remission, even in case of an aggressive resection (30, 31, 35–41).

## 5 Pathology

The pathological classification of pituitary adenoma/PitNETs has been recently remodeled, according to the “2017 WHO Classification of Tumors of Endocrine Organs”, and to a subsequent document by the European Pituitary Pathology Group (EPPG) on pituitary pathology (42, 43), which anticipate the new 2022 WHO Classification (44). The use of the “pituitary neuroendocrine tumors” is still matter of debate. The PANOMEN Workshop recommends that the term adenoma be retained (Ho 2021) and the 5th Edition of the WHO Classification of Endocrine and Neuroendocrine Tumors retains adenoma in duality as transition terminology (Ho 2022). According to the last WHO classification, we use the dual PitNET/adenoma term. A clinical-pathological approach was introduced based on a combination of parameters with important prognostic value, as will be seen when discussing the therapeutic approach. For this reason, in the management of adenomas/PitNET, a complete pathology report is necessary according to the criteria of the pituitary center of excellence (13). Therefore, preoperative information such as clinical and GH/IGF-I plasma levels, as well as MRI features, are mandatory. Moreover, for risk stratification, it is crucial to know the following: histology (mitotic count and histological invasion); immunohistochemistry [pituitary hormone reactivity, cytokeratin pattern (densely or sparsely granulated), proliferation markers (MIB1/Ki-67), p53 percentage, somatostatin receptos (SSTR) type and score (at least SSTR-2 and -5 if requested)] according to IRS (45) or Volante (46) Score. In selected cases, it is necessary to know O6-methylguanine-DNA methyl-transferase (MGMT) status. Unfortunately, there is currently no reliable marker of malignancy. However, the recent validation of the French five-tiered classification, which considers clinical and histological parameters, seems to be a good starting point for the introduction of a better classification of GH-secreting adenomas/PitNETs in terms of aggressiveness (7). In selected patients stratified by a risk category system, the identification of aryl hydrocarbon receptor-interacting protein (AIP) gene mutation can lead to the detection of carriers, potentially leading to a better prognosis (47). Recently, an interesting algorithm have been created to try to predict response to SRL prime line treatment from features such as age at diagnosis, sex, GH, and IGF-I levels at diagnosis and at pretreatment, SSTR-2 and -5 and cytokeratin granulation pattern (48).

## 6 Medical treatment

The effective management of GH-secreting adenomas/PitNETs tumors firstly requires the suppression of autonomous GH secretion, normalization of the IGF-I, and removal or (at least) debulking of the pituitary tumor mass (2).

A convincing and detailed clinical definition of difficult/aggressive GH-adenomas/PitNET was proposed some years ago by the group of Shlomo Melmed (4). Based on cluster analysis, acromegaly patients were stratified into three phenotypes that range from benign (type 1) to aggressive (type 3). Patients with young age at diagnosis, short progressive disease duration, SG invasive macroadenomas, high GH and IGF-I secretion output, high Ki-67, low expression of SSTR2, and who require multiple treatment modalities belong to the latter group.

The large majority of aggressive GH-secreting adenomas/PitNET are characterized by resistance to treatment with first-generation somatostatin analogs. Current definition of resistance to SRL is based on the efficacy to control GH and IGF-I secretion and to induce tumor shrinkage (49). “Biochemical resistance” and a “tumor mass resistance” may be distinguished.

Whereas biochemical and tumoral responses are generally associated, there are some patients in which these responses are discordant (50, 51). The frequency of SRL resistance may also be influenced by the treatment setting, the duration and dosage of treatments, and by the use of optimal GH and IGF-I assay, a part of tumor phenotype (52).

Patients considered partially or completely resistant to first-generation SRL more frequently had post-surgical tumor residual, with higher secretion of GH and IGF-I (53). Tumor proliferation may predict the outcome of medical treatment in acromegaly: lower proliferative index (Ki-67) is typically identified in tumors responsive to first-generation SRL (54). SG GH-secreting tumors are more frequently resistant to SRL in contrast to DG lesions (54, 55). The hyper-intensity of the tumoral mass in T2-weighted MRIs seems to be associated with the SG cytokeratin pattern and resistance to treatment with first-generation SRL (56). Great interest is related to the role of SSTR in predicting the response to SRL (57). Several studies have demonstrated that tumors responsive to first-generation SRL showed diffuse and membranous SSTR2A expression (58, 59). In case of persistent acromegaly after neurosurgery and/or a standard dose of SRL (octreotide LAR 30 mg/28 days or lanreotide autogel 120 mg/28 days), a treatment regimen with higher dose or increased dose frequency compared to a conventional SRL may be useful, in particular in those patients who have reached a certain/partial response to the standard dose of first-generation SRL (60).

The current guidelines suggest the use of Peg-V in patients of irrelevant residual tumor and in those with alterations of glucose metabolism, while treatment with first-generation analogues with Peg-V or PAS should be chosen in the presence of important tumor concerns (60).

With regard of predictors of second-line therapies (Pegvisomant and Pasireotide Lar), a poor response to Peg-V seems related to high pre-treatment levels of GH/IGF-I, large tumor extension, and high Ki-67 values (61), while tumor extension to III ventricle, high pre-treatment GH/IGF-I levels, densely granulated PitNET, low SSTR5 score, complete resistance to first-generation SSA, and high Ki-67 values are related with a PAS poor response (62, 63).

Concerning the adenomas/PitNETs GH/IGF-I secretory output, the pharmacogenomics of the GH receptor seem to play a role: in fact, the presence of the deleted isoform of exon 3 of the receptor (d3-GHR) seems to correlate with a poor response to both pasireotide and to standard dosages of Peg-V (61, 63). Peptide receptor radionuclide therapy (PRRT) seems to be a promising treatment in selected cases, expressing SSTR and demonstrating sufficient tumor uptake of tracer by ^68^Ga-DOTATATE-PET/CT scan (64).

In the rare cases with full absence/low score of SSTR2 and SSTR5 expression, syndromic acromegaly should be investigated. These patients should be treated with Pegvisomant in monotherapy, even in the presence of high proliferative activity and invasive tumors, but an aggressive approach to residual tumor is mandatory, with debulking surgery if clinically appropriate or with radiotherapy/radiosurgery, also in combination with temozolomide (TMZ) (2, 63, 65). TMZ is first-line chemotherapy and recommended for treatment of aggressive adenomas/PitNETs (5, 64) that are resistant to previous therapies.

**Figure 1:**
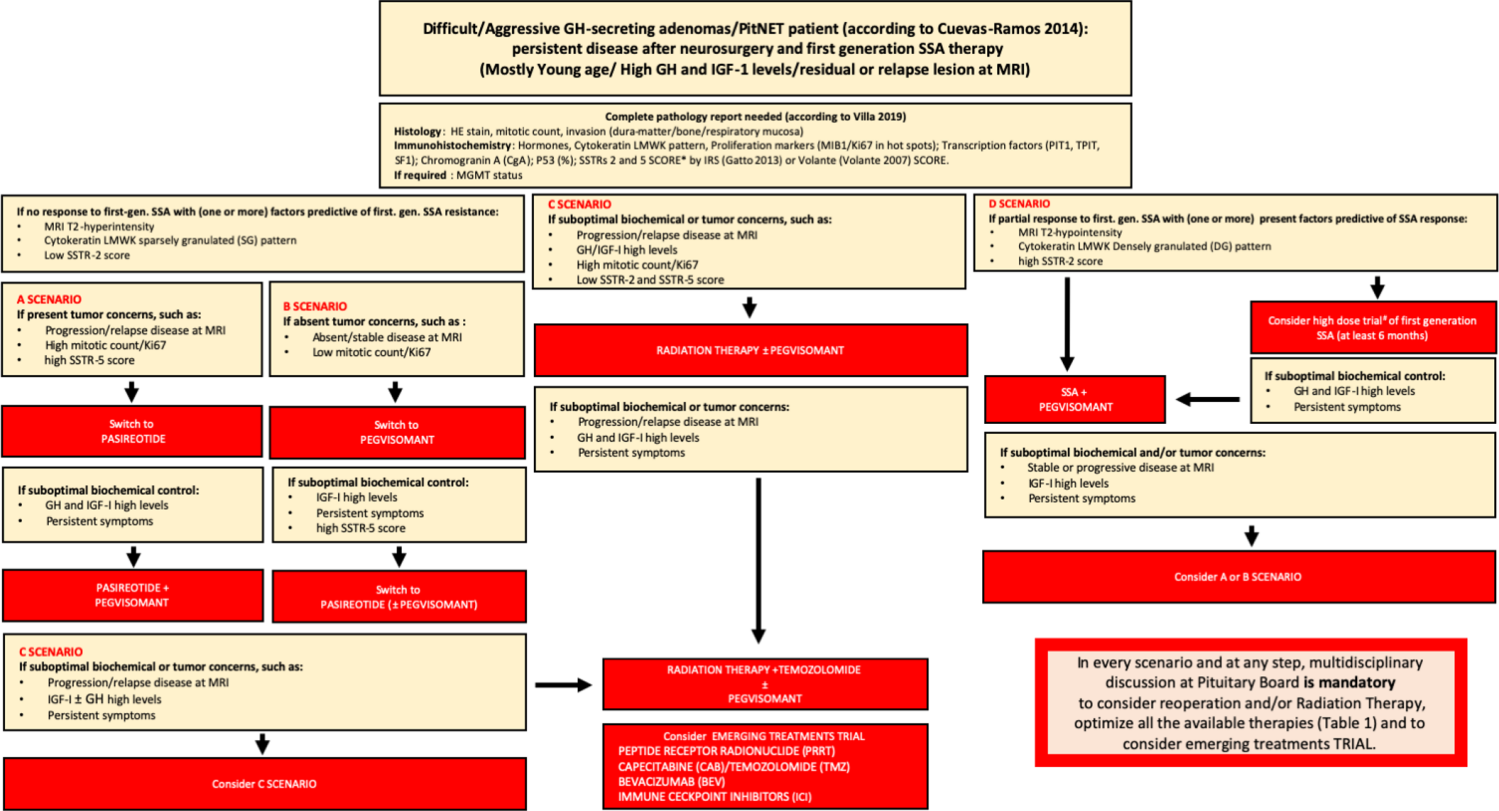
Flow-chart for the management of difficult/aggressive GH-secreting PitNET. After a complete pathology report according to most recent WHO classification, in the presence of no response to first-generation -SSA (and usually one or more factors predictive or first-generation SSA resistance – MRI T2-hyperintensity, cytokeratin SG pattern, low SSTR-2 score), there are 4 scenarios on the basis of tumor concerns and SSTR-5 score: A scenario (in presence of tumor concerns and high SSTR-5 score) drives the switch to PAS treatment alone or, after suboptimal biochemical control, combined to Peg-V; in B scenario (if absent tumor concerns and regardless SSTR-5 score) Peg-V alone is suggested, with the possibility to add PAS if suboptimal biochemical control is present and SSTR-5 score is favorable; in C scenario (in presence of tumor concerns and low SSTR-5 score), Peg-V + Radiation Therapy ± Temozolomide are the main treatment choices. We suggest that patients considered partially resistant to first-generation SSAs may be treated with SSA + Peg-V in cases of expected benign and favorable disease behavior, taking into account a low proliferative index, a non-detectable or not-invasive residual and in the presence of a high SSTR-2 score; alternatively, a high-dose trial of SSA is suggested (D scenario). If after SSA + Peg-V treatment, no biochemical control is achieved, on the basis of tumor concerns and SSTR-5 pattern, A or B scenario is the following step. Finally, if no disease control is obtained after Peg-V + Radiation Therapy ± Temozolomide, we suggest to consider an emerging treatment trial. In every scenario and at every step, multidisciplinary discussion at the Pituitary Board is mandatory to carefully consider reoperation and/or RT and/or every medical treatment choice.* = IRS Score: low expression of SSTRs = score > 6; high expression of SSTRs = score ³ 6; Volante Score: low expression of SSTRs = score 0 - 1; high expression of SSTRs = 2-3; IRS Score; # = lanreotide 120 mg every 14 or 21 days; lanreotide 180 mg every 28 days; or octreotide LAR 30 mg/ every 14 or 21 weeks; octreotide LAR 60 mg every 28 days.

In summary, in **Figure 1**, we report our proposed therapeutic flowchart that is based, after unsuccessfully neurosurgery, on biochemical response to first generation SRL. We consider mandatory the knowledge of GH/IGF-I levels and tumor concerns before starting second line therapies, an molecular biomarkers such as the cytokeratin patterns, the proliferative index, expression of SSTRs We propose four clinical scenarios (**Figure 1**) driving the patient management from the possibility to try a high-dose SRL trial, according to ESE guidelines (in which the therapeutic schedule is monotherapy with first-generation SRL, or Pegvisomant or Pasireotide Lar and combination treatment with first- or second-generation SRL and Pegvisomant (60).

The few patients that fail to reach disease control despite all these multimodal treatments should also be evaluated with the support of an oncologist. In this view, new target therapies are emerging, such as Capecitabine (CAP) or one of its metabolites 5-fluorouraciel (5-FU) (65); Bevacizumab (BEV), and the immune checkpoint inhibitors (ICI) (64, 66). BEV, an anti-vascular endothelial growth factor (VEGF), acts by inhibition of tumor neoangiogenesis. ICI use has been reported in a few cases of aggressive lactotroph and corticotroph adenomas/PitNETs. The effectiveness of this therapy in adenomas/PitNETs is still under investigation in pre-clinical and clinical studies (64, 66). In contrast to aggressive prolactinomas, tumors that express the Epidermal Growth Factor Receptor (EGFR) can be treated with tyrosine kinase inhibitors (TKI) (67). Everolimus (EVE), an inhibitor of the phosphatidylinositol 3-kinase/mammalian target of rapamycin (mTOR) pathway mTOR, approved to treat neuroendocrine tumors, has been demonstrated to be effective in many *in vitro* studies on pituitary cells. However, it showed poor efficacy in the few cases of adenomas/PitNETs reported in the literature, and only two clinical cases have been reported that described acromegaly patients being treated with VEGFR inhibitors. Clinical reports are not available for the treatment of acromegaly patients with mTOR inhibitors, TKI,or ICIs (66). Concerning new emerging drugs, such as oral somatostatin receptors ligands, no data are available on the possible use on aggressive cases. However recent preliminary data on the efficacy of paltusotine on maintain IGF-I at levels comparable to prior injected combination treatment are promising (68).

## 7 Radiation therapy

Radiation therapy (RT) (**Table 1**) is considered when a repeated surgery is not feasible, in case of residual active disease, or after drug therapy failure (76), or in cases with unresectable residual tumor mass. Hormonal normalization appears to be increased at 60.3% of patients if those still on pharmacological therapy after RT therapy are included. The time to reach biochemical remission varies between individual retrospective experiences and depends on pretreatment levels of GH and IGF-1 (83, 84). The choice of irradiation technique should be based on the features of the target tumor. Stereotactic radiosurgery (SRS) is a suitable treatment for patients with relatively small residual adenomas/PitNETs: the proximity to critical structures are limiting factors for its application, with 8 and 10 Gy being the maximum tolerated doses to the optic apparatus (85). Fractionated stereotactic radiation therapy (FSRT) is generally preferred for patients with larger a GH-PitNET that is not susceptible to SRS (85).

**Table 1:** Available treatment tools of difficult/aggressive GH-secreting PitNET.

TOOLS	MECHANISM	EFFICACY	LIMITATIONS/SIDE EFFECTS
NEUROSURGERY (NS)	Physical resection of the tumor	First line NS treatment of difficult/aggressive GH-PitNET is poorly curative, but it can provide tumor debulking, which leads to better responses to adjuvant therapies, as well as histological characterization of the tumor.	True invasiveness remains a major limiting factor for surgical disease remission, even in case of aggressive resection (30, 31, 35–41). Surgical risks might be higher in recurrent cases.
MEDICAL TREATMENT			
*First-generation somatostatin receptor ligands long-acting (SRL)* *(octreotide LAR and lanreotide)*	Suppression of synthesis and secretion of GH through somatostatin receptor 2 on PitNET (1)	In unselected patients, biochemical control in approximately 40% of patients and tumor shrinkage in over 60% of patients (1). In difficult/aggressive acromegaly resistant to standard treatment high, dose trial for at least 6 months is effective in 27.6-36% of patients (69, 70).	Good safety profile at high-dose treatment without no differences with standard regimen in terms of asymptomatic gallstones, nausea, diarrhea, flatulence, FPG and HbA1C (69, 70).
*Pegvisomant (Peg-V)*	Genetically engineered GH analog, with antagonistic effect (71), blocking GH binding and subsequent IGF-I production.	Peg-V is effective in controlling IGF-I levels in 75.4% of patients based on the latest acrostudy update (72).	Favorable safety profile, in terms of liver abnormalities (abnormal AST or ALT in 3.2% of subjects), tumor growth (PitNET increase in 7.1% of patients), lipohypertrophy (1.2%), and decreased IGF1 levels (1.1%) (72). Peg-V improves glucose tolerance and insulin sensitivity in acromegalic patients with blood glucose alterations (71).
*Pasireotide (PAS)*	Suppression of synthesis and secretion of GH through somatostatin receptor 5 (high binding affinity), 2,3 and 1 on adenoma/PitNET tissue (59, 73, 74)	PAS is a multireceptor-targeted SSA, able to bind four of five SSTRs. The rate of biochemical control of acromegaly during treatment with long-acting formulations (Pasireotide Lar) ranged from 14.6% to 93.3% of cases, while the tumor shrinkage is observed in 54.3-80.8% of cases (shrinkage > 20%) or 11-18% of cases (shrinkage <25%). This large range is based on the different criteria applied in studies to define control of disease (74).	The side-effects of PAS are similar to first-generation SRL, including, constipation, diarrhea and asymptomatic cholelithiasis. However, high frequency of glucose metabolism abnormalities are reported (74). It should be noted that in real life, discontinuation of PAS for glycemic imbalance is rare (about 4%) of cases (74) and for the onset/worsening of PAS-related glucose abnormalities the pre-treatment glucose-status is crucial (75).
RADIATION THERAPY (RT*)*	RT can be subdivided into conventional fractionated external beam radiotherapy (EBRT) and single-session or hypofractionated sterotactic radiosurgery (SRS). SRS is delivered using photons (Gamma Knife, CyberKnife, Linear Accelerator) or proton beam therapy (76, 77)	In EBRT, the total dose of 45-55 Gray (Gy) is obtained with daily doses of 1.8-2.0 Gy, with a treatment course of 6 weeks. SRS delivers treatment doses (18-32 Gy) in one shot or in 3-5 sessions (Fractioned Stereotactic Radiosurgery, FSRS). Biochemical remission rates after EBRT occurs in about 50-60% of patients, with up to 90% of patients with tumor control at 10 years. Biochemical remission after SRS occurs in 48-53% of patients, with up to 95% of patients having tumor control at 10 years (77–80)	The main post-treatment effect is hypopituitarism, described in 15-50% of cases (nearly 30% for SRS and 50% for EBRT). Visual impairment (0-5%), radiation associated secondary intracranial tumors (0-2%), and neurocognitive deficits (up to 21% after 20 years of follow up) are more frequent in EBRT than SRS (80). Visual disturbances after SRS treatment are rare and depend on the proximity of the tumor to the optic pathways (cut-off: 3 mm) and the dose (77, 79). A recent study showed that Gamma Knife radiosurgery (GK) does not seem to induce long- term cognitive consequences (81)
Temozolomide (TMZ)	Temozolomide (TMZ) is an alkylating agent that under- goes rapid chemical conversion in the systemic circulation at physiological pH to the active compound [5-(3-methyl- triazeno) imidazole-4 carboxamide] (82)	TMZ standard dose of 150-200 mg/m^2^ for 5 consecutive days every 28 for at least 5 cycles shows and immediate response both in terms of tumor burden and secretory output in about 40% of patients, and is usually well-tolerated (64, 66)	Fatigue and nausea are the most common side effects, but rarely different degrees of bone marrow affections were observed, from low granulocyte count to severe depression with pancytopenia (64)

## 8 Nuclear medicine

In patients with GH-PitNET, positron emission tomography-computed tomography (PET-CT) with ^18^F-fluorodeoxyglucose (FDG PET-CT) has been shown to be helpful for preoperative characterization of sellar lesions, but with conflicting results (86–90). Other tracers have been developed and clinically validated in this setting, such as ^68^Ga-labeled somatostatin analogues (specifically, DOTATOC, DOTANOC and DOTATATE) and ^11^carbon-methionine (C-MET). ^68^Ga-labeled somatostatin analogues show high specificity in binding to SSTRs 2, 3 and 5, which are expressed by normal pituitary tissue and are hyperexpressed by GH-secreting adenoma/PitNET (91). Although in most patients, even if treatment-naïve, adenoma/PitNETs show significantly lower ^68^Ga-DOTATOC uptake compared to the normal pituitary gland, SSTR expression on the surface of PitNET cells (immuno-histochemically proven “a posteriori”) may lead to increased uptake in the adenoma/PitNET, which can be useful in localizing and determining response to surgical, medical, or radiation therapy. In particular, the expression of SSTRs and therefore the SUVmax is higher in patients with high ^68^Ga-DOTATOC uptake lesions (92); it may help to detect possible surgical failure (93); it is significantly and inversely correlated with lower circulating GH levels and complete laboratory response (94). ^68^Ga-labeled SRL are helpful in selecting patients who are suitable for peptide receptor radionuclide therapy (PRRT) which consists of administrating a therapeutic dosage of beta-emitting somatostatin analogue in order to carry lethal radiation to target cells: currently, ^177^Lu- and ^90^Y-labeled compounds are available for this purpose. PRRT is a novel promising treatment in patients with extensive and aggressive adenoma/PitNETs who are not suitable for surgery or refractory to medical/external radiation treatment, manifesting lower systemic adverse effects than conventional external beam radiation due to its targeted nature (67). PRRT has been used in 15 cases from 2012 to 2020 in patients with aggressive adenoma/PitNETs and carcinomas, either functioning or non-functioning, with varied radiopharmaceuticals, protocols and measured outcomes, making generalization on its effectiveness in this setting not feasible (66); only 43% patients had responded to PRRT, but most non-responders were resistant to previous temozolomide treatment and therefore had a more aggressive disease, such as a case of aggressive GH-secreting adenoma/PitNET completely resistant to other treatments and successfully treated with ^90^Y-DOTATATE (95). Overall, PRRT is well tolerated since it does not carry a higher risk of developing hypopituitarism (96, 97). C-MET PET-CT also plays an interesting role in diagnosing recurrent adenoma/PitNETs in patients already treated with adenomectomy or sub-total hypophysectomy, due to its ability to collect into cells with an increased amino acid intake and protein synthesis/secretion. Different from ^68^Ga-labeled somatostatin analogues, C-MET uptake is not affected by SSTRs expression or hormone secretion. For these reasons, in patients operated on for an aggressive GH-adenoma/PitNET with persistent or relapsed disease and with equivocal MRI, it is a promising tool to drive and facilitate neurosurgeons to perform targeted revision surgery (98).

## 9 Open issues and conclusions

In tertiary care pituitary centers, difficult/aggressive GH-secreting adenomas/PitNET might be more frequent than usually encountered in a primary endocrinology clinic, justifying the definition and recognition as adenomas/PitNET. A synthesis between the different positions is absolutely needed and desirable. The new WHO 2022 should be implemented with a structured pathology report, which should be the cornerstone to investigate a staging system. In our opinion, a complete pathology report is mandatory for the definition of a Pituitary Center of Excellence, together with the ability to offer a multidisciplinary approach. Recent papers have described the prognostic role on known clinical and molecular markers such as proliferation index, granulation, or SSTR expression pattern (6, 99, 100). Nonetheless, pathological analysis needs to be still standardized and validated before being included in future guidelines. In this regard, it is necessary to implement research not only on these and new prognostic factors of tumor biology and of the tumor immune microenvironment, but on the pathophysiology of GH secretion, which may represent a target for future molecular therapies (7, 101, 102). PET-CT could play a role in the diagnosis and treatment of pituitary adenoma/PitNET, and GH-secreting specifically ones, for detecting persistent or refractory disease and for selecting patients who are suitable for third-line therapies such as PRRT. In this regard, joint action of an experienced multidisciplinary team is required to progress and improve the management of acromegaly patients with difficult/aggressive GH-secreting adenomas/PitNET.

## Author contributions

AB, SC, AG, SG, CM, CC, MR, MG, LL, FD wrote the manuscipt. LM, AO, AP reviewed the manuscript. All authors contributed to the article and approved the submitted version. 
